# Enterprise resource planning implementation within science and technology park (STP) organisations: an avenue for future research. A systematic review.

**DOI:** 10.12688/f1000research.73347.3

**Published:** 2023-08-10

**Authors:** Sharbani Harun, Magiswary Dorasamy, Abdul Aziz Bin Ahmad, Ching Seng Yap, Saida Harguem

**Affiliations:** 1 Faculty of Management, Multimedia University, Cyberjaya, Selangor, 63100, Malaysia; 2 Technology Park Malaysia, Kuala Lumpur, Wilayah Persekutuan, 57000, Malaysia; 3 Curtin University Malaysian, Miri, Sarawak, 98009, Malaysia; 4 Canadian University Dubai, Dubai, United Arab Emirates

**Keywords:** Enterprise Resource Planning (ERP), Organisational Performance, Accelerated ASAP, Science & Technology Park (STP), Post implementation, Malaysia, End User Behaviour, Systematic Literature Review

## Abstract

**Background:** Enterprise resource planning (ERP) is critical to enhancing the ability to control commercial activities and results in a competitive advantage when combined with an organisation's existing competitive advantages. However, our practise review reveals that end users resist ERP implementation because the resulting changes will alter the current status quo. The implementation of an ERP system in an organisation is complex as it affects multiple areas of the business. Resistance to change is cited as a factor of ERP failure.

**Methods:** In this study, we conducted a systematic literature review using Transfield’s five stages and established a conceptual framework for ERP system implementation in science and technology parks (STPs). Articles collected from Emerald, Science Direct, ProQuest and Scopus databases between 1 ^st^ June 2021 and 15 ^th^ June 2021. Two authors were assigned to check the suitability of the articles in order to avoid risk of bias. Articles were analysed based on components of a research paper and the data was tabulated using MS Excel.

**Results:** Only eight papers (0.011% of all the papers) appeared when we searched for papers related to ERP with a focus on post ERP Implementation, end-user behaviours, organisational performance, and the accelerated SAP (system application and product) methodology. We found that there are hardly any articles on ERP post implementations in STP context particularly based on the evaluation part of accelerated SAP.

**Conclusions:** Results indicate the lack of studies in this field, particularly those addressing issues related to STP. This study attempted to broaden the understanding of the ERP's effectiveness, particularly in terms of an organisation's operational performance.

## Introduction

Organisations must improve their own business practices and procedures to survive in a rapidly changing business environment. Enterprise resource planning (ERP) systems are the most significant development incorporating information technology use and are quickly becoming the backbone of organisations. The challenges and high failure rates associated with ERP implementation have been extensively discussed in the literature. Companies typically experience business problems after investing a significant amount of money in an ERP system but are inevitably left with an ERP investment for which they received nothing in return. ^1^ In terms of ERP implementation, one of Malaysia's selected science and technology parks (STPs) is in a similar situation. End-users frequently object to ERP implementations due to the inevitable changes in their current work environment. ^2^ According to a study, ERP implementations fail because employees cannot articulate and meet their changing expectations. ^3^ The acceptance or rejection of technological change has long been recognised as a factor in a person's ability to enhance performance and productivity, while low productivity and dissatisfied customers may result from the unfavourable attitude of employees towards ERP implementation. In addition, excessive organisational spending can lead to a negative view of the ERP system among stakeholders. The selected STP organisation for this study decided to continue implementing ERP based on an assessment of the benefits and challenges of the implementation because it is an important component of their digital transformation initiative. The non-financial and financial benefits were used to justify the organisation's decision to implement ERP. After years of implementing the ERP system in the selected STP in Malaysia, it is still unsure whether the anticipated benefits, such as improved operational performance and the value-added through the integration of business processes with best practices, have been realised as no evaluation of the ERP system's post-implementation benefits has been conducted. From the preliminary interviews with end-users in the STP organisation, mixed reactions to the use of ERP, both positive and negative, can be reasonably assumed.

The most difficult phase of an ERP project is the implementation. ^4^ Most leading ERP providers, such as SAP (system application and product), have their own implementation methodologies. The accelerated SAP (ASAP) methodology was created by SAP. ASAP ^5^ is a methodology for planning, managing, and delivering SAP efficiently and cost-effectively. However, as the ERP lifecycle has no evaluation component, the benefits of implementing and using ERP for the selected STP could not be evaluated. Thus, after completing the ERP implementation project, these evaluation elements may be incorporated into ASAP, demonstrating the benefits of ERP. This study will eventually lead to the development of a framework that incorporates evaluation elements into the post ERP implementation phases of the ASAP methodology. Therefore, this study aims to analyse the factors that contribute to user resistance and provide necessary information for decision-makers to complete the actions required to achieve success in the case of STP.

### Challenges in ERP implementation

ERPs are critical tools for acquiring the capability to control commercial activities and result in a competitive advantage when combined with a firm’s existing competitive advantages. ^6^ A study recommended several strategies for maximising the ERP system’s benefits and thus improving the overall organisational performance. ^7^ Businesses primarily implement ERP to streamline operations, improve performance and create value by integrating business processes with best practices, management functions, real-time reporting, and the ability to study data. An ERP system enables a business to holistically manage all aspects of its operations to remain competitive in the current business environment. ^8^

ERP systems enable companies to handle their operations effectively with possible benefits, including increased process efficiency, improved data analysis, increased consistency in decision-making, lean inventory, improved communication across the supply chain, and strong customer experience. ^9^ Furthermore, ERP has resulted in substantial cost savings, increased return on investment, and improved access to information, ultimately improving consumer decision-making. ^10^ In addition, ERP increases productivity, decreases downtime, reduces lead times, decreases the order cycle time, minimises inventory, reduces the system installation time, reduces quality issues, decreases the scrap rate, decreases the rework rate, and increases the overall company productivity and pay. ^11^ According to studies, ERP systems can also help an organisation increase its capacity and flexibility in response to changing client needs. ^12^

Companies usually have business problems after investing a significant amount of money in an ERP system but end up with an ERP investment for which they have not received anything in return. ^13^ In terms of ERP implementation, the STP in Malaysia selected for this study is in a similar situation. Following the implementation of ERP in the selected STP, the lack of end-user support in the system’s use and the absence of organisation performance evaluation were observed. Understanding critical issues will aid organisations in the successful carrying out of ERP systems in the future. Managers and those responsible for information must also understand and quantify the benefits of the ERP system to justify ongoing costs and the impacts to the organisation. ^14^ Generally, the impacts and benefits of implementing an ERP system are not realised until the system has been completed and is fully operational.

### Relevant theories for ERP implementation

According to the literature, several theories can shed light on the challenges mentioned above for STP context in the Table 1. These theories were obtained from preliminary reading prior to the systematic review.

**Table 1.** Relevant theories. ERP = enterprise resource planning.

Theories	Describing the theories	Why this theory is suitable
Social Technical System (STS) Theory	The STS was developed to emphasize the critical role of interactions between individuals and processes in achieving organizational goals. ^15^	This STS theory suggested that combining social and technological solutions in the implementation of ERP can enhance performance. Under the STS theory, ERP performance is improved when the system is aligned to operational and social needs. User participation increases user acceptance of the system while reducing the probability of resistance. ^16^
Balance Scorecard (BSC)	The Balance Scorecard (BSC) developed by Kaplan and Norton (1992) can be used to manage the overall assessment of a company's performance as well as to align the company's vision and strategies.	The researcher enthusiastically endorsed the Balanced Scorecard as the appropriate assessment mechanism for ERP system implementation investment projects. ^17^ The researcher confirmed that BSC addresses exactly two main tasks of ERP management. First, the BSC helps turn visions into strategies and, finally, into an ongoing business that meets business goals. Secondly, ongoing monitoring and control of the system's operation is required to optimise its use. ^18^
Unified Theory of Acceptance and Use of Technology (UTAUT)	The UTAUT model (Venkatesh, 2003) evaluates the probability of successful implementation of the ERP by giving an overview of the degree of user willingness to accept the technology. ^20^	The UTAUT model can assist organizations in identifying and comprehending the factors that influence acceptance, allowing for proactive design and facilitating actions such as marketing and training directed at those identified as less likely to adapt, adopt, and use ERP systems. ^19^ As a result, the UTAUT model will be used in this study to help gather underpinning data that will aid in identifying factors and appropriateness toward influencing the ERP effective use and return of investment (ROI).

While there are other theories such as TAM, DOI, and RBV that could potentially be relevant to ERP implementation study, our initial reading led us to focus on Sociotechnical system theory, UTAUT, and BSC due to their strong alignment with the specific context of Science and Technology Parks (STPs) and ERP implementation. The novelty of this research lies in the context of this study which is STP. These theories are aligned to lead to most pertinent insights and direction for the research objectives of an empirical study following this SLR paper.

The objectives of this proposal are as follows:

a) To analyse the research gaps on the implementation of ERP in the STP organisation in Malaysia
b) To propose a conceptual framework for the STP based on the literature review

## Methods

### Ethical considerations

This study was approved by the Research Ethical Committee (REC) of Multimedia University (EA2722021). This article is reported in line with the Preferred Reporting Items for Systematic Reviews and Meta-Analyses (PRISMA) guidelines. ^30^

### Study design

This is a systematic literature review paper that highlights the challenges faced by STPs regarding ERP. Thus, we aim to present a preliminary conceptual framework that the body of knowledge in the field of ERP can adopt in STPs. To bridge the research gap in this field, we searched the current research landscape using Tranfield’s five stages. ^21^ Tranfield's method is a well-known and highly cited method for carrying out systematic reviews of the literature. Our literature review was based on the following five stages:

a) Planning the review
b) Identifying and evaluating studies
c) Extracting and synthesising data
d) Reporting descriptive findings
e) Utilising the findings to inform research and practice

### Stage 1: Planning the review

The primary objective of this review is to ascertain the nature and scope of the research conducted on the selected STP’s ERP implementation and subsequent organisational performance. We intend to provide researchers with a comprehensive review of post ERP implementation in STP, with particular emphasis on end-users who oppose ERP implementation due to the disruption of the current status quo. The objective is to provide information on the hidden causes of such behavioural change and inform the knowledge community about potential research directions for the field.

### Stage 2: Identifying and evaluating studies

This study aims to identify solutions that will enable managers to make more informed decisions, mitigate the impact of technological change on the organisation’s social system, significantly reduce the implementation time, increase manager training and balance the effectiveness of ERP in manufacturing firms. ^22^ The knowledge gained from this research might have broad implications for raising managers’ and employees’ awareness of the critical nature of preparation before implementing ERP systems and technical change. ^23^

This study aims to examine the organisational benefits of ERP after implementation and their impact on the organisational efficiency and operational performance of a Malaysian STP. This study's objectives include determining the ERP system's post-implementation benefits and its effective use in Malaysia’s STP. The researchers believe that understanding all the shortfalls is critical to the full comprehension of the consequences of ERP implementation in the Malaysian STP. Additionally, this research aims to develop an ERP framework for future use in Malaysian STP organisations to maximise the benefits of post-ERP implementation.

*Inclusion and exclusion criteria*

Articles were collected from database inception to 15 June 2021. Table 2 shows the inclusion and exclusion criteria of the articles. The search was conducted between 1 June 2021 – 15 June 2021.

**Table 2.** Inclusion and exclusion criteria.

Criterion	Inclusion	Exclusion
Literature type	Indexed journal (Research Articles)	Non-indexed journals, systematic review articles, chapters in book, conference proceedings
Language	English	Other languages
Indexes	Scopus, Social Science Citation Index (SSCI), Australian Business Deans Council (ABDC) List, and Education Research Abstracts Online (ERA).	Non-indexed journals

*Search strategy*

We searched four major online databases ( Emerald, Scopus, Pro Quest, Science Direct) as they are largest databases for social science journal articles. The following keywords were used: (1) enterprise resource planning (ERP), (2) ERP AND post-implementation, (3) ERP AND post-implementation AND end-user behaviours, and (4) ERP AND post-implementation AND end-user behaviours AND organisational performance AND Accelerated SAP (ASAP) methodology. The keywords set used in this study is provided in Table 3.

**Table 3.** Search results.

	Keyword combination used				
Database online	Enterprise Resource Planning (ERP)	Enterprise Resource Planning (ERP)	Enterprise Resource Planning (ERP)	Enterprise Resource Planning (ERP)	Enterprise Resource Planning (ERP)
		AND	AND	AND	AND
		Post Implementation	Post Implementation	Post Implementation	Post Implementation
			AND	AND	AND
			End-User Behaviours	End-User Behaviours	End-User Behaviours
				AND	AND
				Organizational Performance	Organizational Performance
					AND
					ASAP
Emerald	6,000	387	133	131	2
Scopus	39,191	264	4	1	0
Pro Quest	15,275	138	30	30	1
Science Direct	12,169	2,400	407	362	5
Total	72,635	3,189	574	524	8

### Stage 3: Extracting and synthesising data

Two authors collected the papers together collaboratively. We collected data related to findings and themes. Stages 4 and 5 of Tranfield are combined and presented in the following sections.

## Results

**Figure 1.**
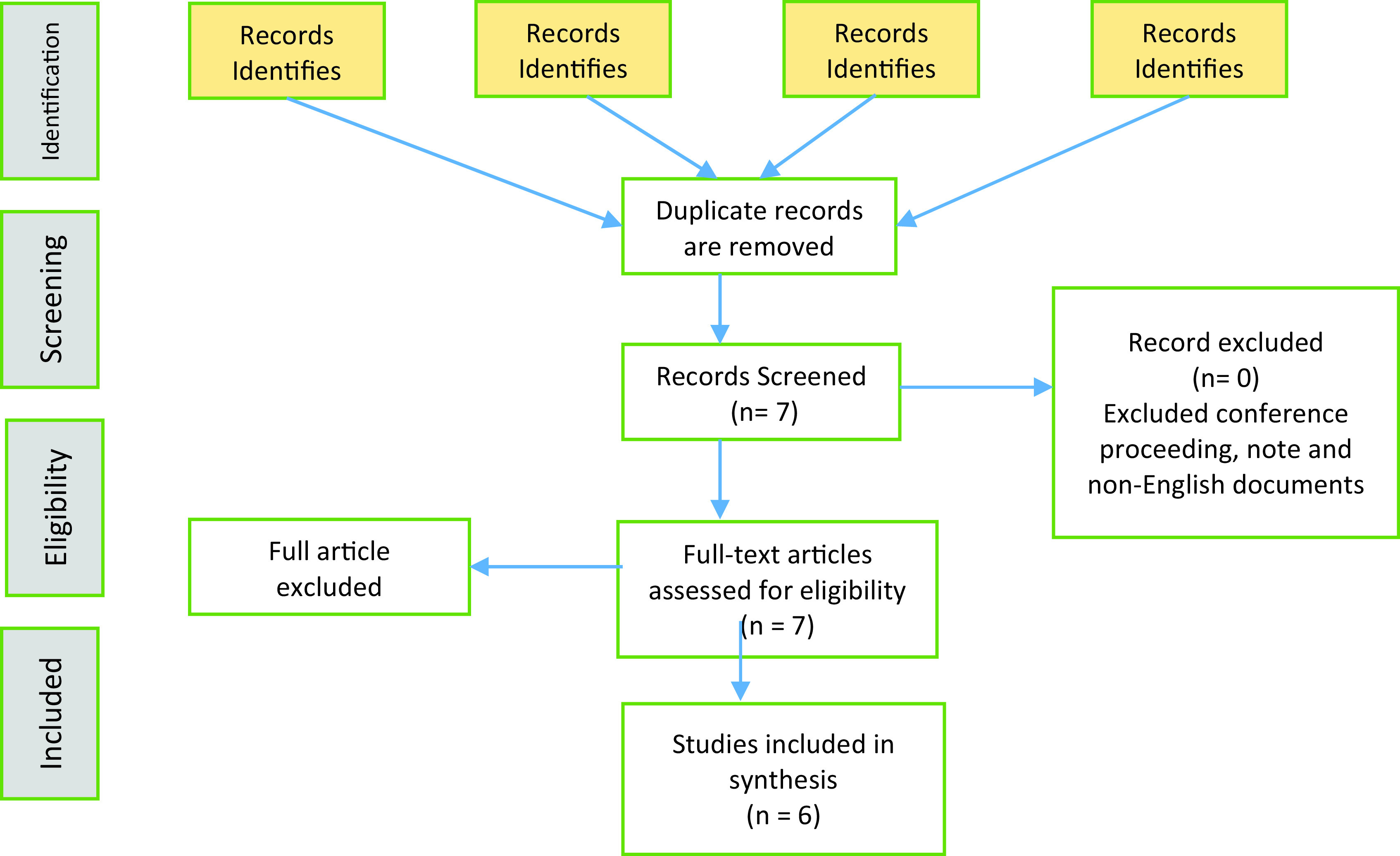
PRISMA Flow Diagram.

A PRISMA flow diagram as shown in Figure 1 illustrates the flow of paper extractions based on the inclusion and exclusion criteria. The number of papers included in this review are presented in Table 3.

### Study selection

The number of articles found per database and search strategy is presented in Table 3. The Scopus database recorded the highest number of ERP papers. When the search was executed with many keywords combined, Science Direct showed more related papers than the other online databases. We found only eight papers when the search for the final combination of ERP, post-implementation, end-user behaviours, organisational performance, and ASAP was executed. The fraction of papers are shown in Table 4. We further analysed the eight papers to gain some insights. Two of the papers were found to be unrelated papers. Hence, only six papers were included in this analysis, as shown in Table 5.

**Table 4.** Fraction of papers by a combination of keywords.

Keywords	Paper count	Percentage
**Enterprise resource planning (ERP)**	72635	100%
**Combination:** **ERP** **Post Implementation**	3189	4.39%
**Combination:** **ERP** **Post Implementation** **End user behaviours,**	574	0.79%
**Combination:** **ERP** **Post Implementation** **End user behaviours, Organizational performance**	524	0.72%
**Combination:** **ERP** **Post Implementation** **End user behaviours, Organizational performance** **ASAP**	8	0.011%

**Table 5.** Synthesis of literature on enterprise resource planning (ERP). CSF=critical success factor.

No	Authors (Year)	Titles of Articles	Findings/Themes
**1**	^24^ Guido & Pierluigi (2009)	A planned-oriented approach for ERP implementation strategy selection	• This methodological approach has been put to the test by running a validation experiment with the company partner of the research project. • Analyzing results showed that the methodology was an effective way of determining the capabilities that firms should have in order to determine the most appropriate implementation strategy.
**2**	^25^ Annamalai & Ramayah (2012)	Does the organizational culture act as a moderator in Indian Enterprise Resource Planning (ERP) projects? An empirical study	• The findings of this study show that organisational culture functions as a moderator and moderates the relationship between CSF and ERP project implementation success in India.
**3**	^26^ Nazeer & Joseph (2017)	Conceptualising a multidimensional model of information communication and technology project complexity	• This article emphasised five factors that influence ICT projects: the likelihood of success, the duration of the project, the complexity of the project, the types of projects, and the methods by which projects are completed. • The dimensions were investigated in order to identify the critical constructs and elements that needed to be considered. The various dimensions were plotted using a multidimensional model.
**4**	^27^ Vinod, Bharat & Uma (2003)	An investigation of critical management issues in ERP implementation: emperical evidence from Canadian organizations	• Despite the fact that each adopting organisation has its own set of goals for its systems project, we discovered many similarities in motivations, concerns, and strategies across organisations. • This study identifies several critical issues in ERP project management.
**5**	^28^ Marina & Neil (2001)	The implementation of Enterprise Resource Planning packages in different organisational and national cultures	• The findings show that there is an association between organisational culture and ERP implementation problems, but there is no direct evidence that there is an association between national culture and implementation problems. Furthermore, the findings show that these various implementation issues can be caused by a misalignment between a small set of core values indicative of a customer's organisational culture. • At the end of the paper, we review our predictions, draw conclusions about them and the work of the key authors of national and organisational culture, and discuss future work.
**6**	^29^ Stanley, Gardiner, Hanna, and La Tour (2002)	ERP and the reengineering of industrial marketing processes: A prescriptive overview for the new-age marketing manager	• ERP systems are related to business engineering (BE), which places emphasis on decades of rigorous research that seeks to establish the most efficient business practises. • As a part of the overview of ERP strategic applications for industrial marketing, there is also a description of the various cases used for applications. For instance, an effective streamlined sales order process is showcased, and the managerial implications are also identified.

Table 4 shows the results based on all the sources and groups of keywords mentioned above. When the ‘ERP’ was used, 72635 articles were discovered. This figure dropped to 3,189 when we used ‘ERP + post implementation’. When we used the keywords ‘ERP + post-implementation + end-user behaviours + organisational performance’, 524 articles were produced. Following the careful selection based on the inclusion and exclusion criteria described in the following section, eight papers about ‘ERP + post-implementation + end-user behaviours + organisational performance + Accelerated SAP (ASAP)’ were identified.

This study identifies several critical issues in the ERP project as illustrated in Table 5. The research reveals that the organisation’s motivations, concerns, and strategies shared many similarities. The findings indicate that organisational culture has an impact on the relationship between the critical success factor (CSF) and the ERP project’s success. Nonetheless, no direct connection seems to exist between national culture and implementation issues.

## Discussion

There are several gaps identified in this study. There are limited studies on post ERP implementation success in the context of STP focusing on end-user behaviour and organisational performance using a balance scorecard approach. This limitation is exhibited in terms of lack of evidence and study on STP particular case studies. The case studies are crucial for STPs to understand and adopt any possible recommendations. Implication of this study contributes to the decision of STP to invest in ERP in order to fully harvest the benefits.

A limitation of this study is the number of keywords selected. Keyword selections are based on the research focus. However, it is possible to obtain more articles if the keywords are expanded to a field of study that is not specific in nature, such as STP. This could possibly lead to publication biases.

### Research gap

Gap 1: The result presents a clear research gap in post-ERP implementation success in the context of the STP that focuses on organisational performance using the balanced scorecard approach.

Gap 2: A research gap in post ERP implementation success exists in the context of the STP that focuses on end-user behaviour to prevent resistance to change.

Gap 3: A research gap exists in terms of the post ERP implementation component in the ASAP methodology, i.e. evaluating the post-implementation is crucial to ERP success.

### Framing the concept

**Figure 2.**
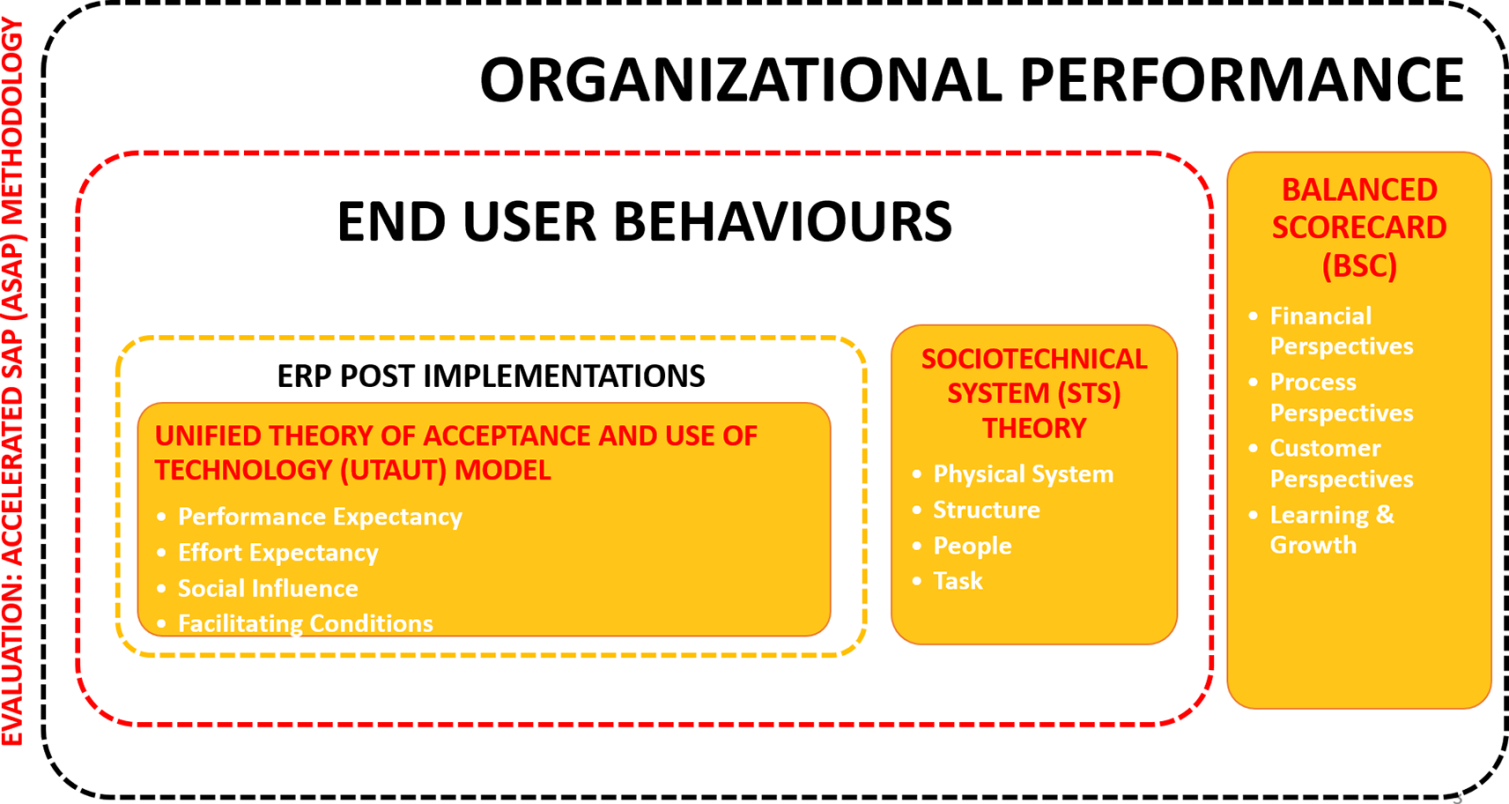
Conceptual framework. ERP=enterprise resource planning; ASAP=Accelerated SAP (System Application and Product).

We propose a conceptual framework in Figure 2 for studies to improve the post-implementation success by focusing on the ASAP methodology to focus on the evaluation stage using two main theories and BSC approach to understand three different dimensions:

1. End-user behaviour: Sociotechnical system theory
2. ERP implementation: Unified theory of acceptance and use of technology model
3. Organisational performance: Balanced scorecard (BSC) approach

### Future research recommendations

Researchers involved in examining the relationship between post ERP implementation success and STP could consider the main findings of the current study to explore options for future work. The two major themes for future research consideration include the following:

• Theme 1: Post ERP implementation evaluation using BSC to evaluate organisation performance
• Theme 2: ERP end-user behaviour towards resistance to changes

## Conclusions

This study aims to inform the ERP for STP knowledge community about the research gaps in the application of theories in post ERP implementation success and organisation performance based on the published literature. In this study, we applied the five-stage methodology of Tranfield et al. (2003) in writing papers based on the comprehensive review of the literature in a given area. This methodology was used to understand the extent and nature of post ERP implementation in STP research in organisational performance. From the extensive search of 72,635 papers in the ERP domain, our search list was narrowed to eight papers (0.011%) that examined the post ERP implementation involving STPs, end-user behaviour, organisational performance, and the ASAP methodology. Our review of the eight papers suggests that the scope for more significant research on the two major areas is present. More empirical work is required to improve the understanding of the determinants of post ERP implementation success factors in the context of STPs. The implication for organisations is that they will have an improved plan in place to harvest their ERP investment in the future.

## Data availability

### Underlying data

All data underlying the results are available as part of the article and no additional source data are required.

### Reporting guidelines

Figshare: PRISMA checklist for ‘Enterprise resource planning implementation within science and technology park (STP) organisations: an avenue for future research. A systematic review’. https://doi.org/10.6084/m9.figshare.16821982. ^30^

Data are available under the terms of the Creative Commons Attribution 4.0 International license (CC-BY 4.0).
