# Supplementary material for: Enterprise resource planning implementation within science and technology park (STP) organisations: an avenue for future research. A systematic review
Source: F1000Res. 2023 Aug 10;10:1148. Originally published 2021 Nov 12. [Version 3] doi: 10.12688/f1000research.73347.3 (PMC10432887; doi:10.12688/f1000research.73347.3)
Supplement: Peer review history

# Peer review: Reviewer response for version 3

**DOI:** 10.5256/f1000research.154401.r195615

**Published:** 2023-08-16

**Authors:** Olakunle Jayeola

**Affiliations:** 1 Adekunle Ajasin University, Akungba, Ondo, Nigeria

The authors gave a good and valid response to my suggestions. Courteous and highly professional in their responses. I agree with them and approve the publication of the article.

Are the rationale for, and objectives of, the Systematic Review clearly stated?

Yes

Is the statistical analysis and its interpretation appropriate?

Yes

Are sufficient details of the methods and analysis provided to allow replication by others?

Yes

Are the conclusions drawn adequately supported by the results presented in the review?

Yes

Reviewer Expertise:

Technology management, ERP,  Cloud computing, Entrepreneurship, and general management

I confirm that I have read this submission and believe that I have an appropriate level of expertise to confirm that it is of an acceptable scientific standard.

# Peer review: Reviewer response for version 2

**DOI:** 10.5256/f1000research.122731.r180974

**Published:** 2023-07-25

**Authors:** Olakunle Jayeola

**Affiliations:** 1 Adekunle Ajasin University, Akungba, Ondo, Nigeria

Minor

- Enterprise resource planning (ERP) is a well-rounded technology that streamlines the activities of a firm for seamless operations and a competitive advantage. Hence, this article addresses gaps/issues concerning ERP and provides a framework for successful implementation in a less-researched context (Science and technology park), contributing significantly to the literature. More importantly, the issue of realising value from ERP implementation which has been a major discourse in the literature is addressed, giving the article its relevance.
- The paper is well-written. The objectives and rationale for the systematic review were spelled out and achieved. The methodology is clear and replicable, especially using PRIMA. The conceptual framework developed for studies to improve post-implementation success by focusing on the ASAP methodology is of great value, especially in the STP context. The conclusion is good.

Major

- However, the authors had a narrow view of the applicable theories. I expected them to review some theories such as TAM, DOI, RBV, etc., and justify why Sociotechnical system theory, UTAUT, and BSC were better. It will be of immense value if authors could incorporate this suggestion. Nevertheless, the paper is well-structured and delivered.

Are the rationale for, and objectives of, the Systematic Review clearly stated?

Yes

Is the statistical analysis and its interpretation appropriate?

Yes

Are sufficient details of the methods and analysis provided to allow replication by others?

Yes

Are the conclusions drawn adequately supported by the results presented in the review?

Yes

Reviewer Expertise:

Technology management, ERP,  Cloud computing, Entrepreneurship, and general management

I confirm that I have read this submission and believe that I have an appropriate level of expertise to confirm that it is of an acceptable scientific standard, however I have significant reservations, as outlined above.

# Response

**Published:** 2023-08-08

**Authors:** Magiswary Dorasamy

**Affiliations:** MULTIMEDIA UNIVERSITY, Malaysia

Thank you for your feedback and acknowledgment of the importance of our article. We appreciate the acknowledgment of how the article addresses ERP gaps in the Science and Technology Park context, significantly contributing to the literature. We agree that focusing on value realization from ERP implementation and developing a clear and replicable methodology using PRIMA are critical aspects of our research. We are pleased that you find the conceptual framework useful for improving post-implementation success, particularly in the STP context. Your comments are encouraging and motivate us to continue researching in this area. Thank you for your feedback and acknowledgment of the importance of our article. We appreciate the acknowledgment of how the article addresses ERP gaps in the Science and Technology Park context, significantly contributing to the literature. We all agree that focusing on value realization from ERP implementation and developing a clear and replicable methodology using PRIMA are critical aspects of our research. We are pleased that you find the conceptual framework useful for improving post-implementation success, particularly in the STP context. Your comments are encouraging and motivate us to continue researching in this area.

 While we appreciate your recommendation to investigate additional theories such as TAM, DOI, and RBV, our reading led us to focus on Sociotechnical system theory, UTAUT, and BSC due to their strong alignment with the specific context of Science and Technology Parks (STPs) and ERP implementation. However, we have included a paragraph to acknowledge these theories in the article. We will consider incorporating a brief justification for our theory choice in future revisions. We are pleased that you found the paper to be well-organized and presented. Your feedback assists us in enhancing the caliber of our work.

# Peer review: Reviewer response for version 2

**DOI:** 10.5256/f1000research.122731.r129076

**Published:** 2022-04-27

**Authors:** Amizawati Mohd Amir

**Affiliations:** 1 Centre for Governance Resilience and Accountability (GRACE), Faculty of Economics and Management, Universiti Kebangsaan Malaysia, Selangor, Malaysia

The authors have revised and responded to the earlier comments. A systematic review of prior work clarifies the gap in the existing knowledge that the study proposes to investigate further. The method to pursue the research process is also being revised.

Are the rationale for, and objectives of, the Systematic Review clearly stated?

Partly

Is the statistical analysis and its interpretation appropriate?

Not applicable

Are sufficient details of the methods and analysis provided to allow replication by others?

Partly

Are the conclusions drawn adequately supported by the results presented in the review?

Partly

Reviewer Expertise:

Management Accounting and Control System

I confirm that I have read this submission and believe that I have an appropriate level of expertise to confirm that it is of an acceptable scientific standard.

# Peer review: Reviewer response for version 1

**DOI:** 10.5256/f1000research.76992.r121593

**Published:** 2022-02-08

**Authors:** Amizawati Mohd Amir

**Affiliations:** 1 Centre for Governance Resilience and Accountability (GRACE), Faculty of Economics and Management, Universiti Kebangsaan Malaysia, Selangor, Malaysia

The aim of the study is to understand that ERP adoption in STP organisations is a current research topic. However, the justification for the motivation of the study is unclear. Given that the ERP has a long development history starting back in the 1990s, what aspect of ERP is the study focusing on? The ERP concept itself has a very broad term and definition, coming from various perspectives and fields of knowledge, hence the focus of ERP itself needs to be clarified. There are various success factors related to ERP adoption, such as technology challenges, human factors, and financial constraints. Thus, a systematic review of prior research is pertinent to highlight the significance of the proposed study.

 The paper states high failure reported on ERP implementation, critical discussion on past evidence contributing to the failure may highlight the challenges which could be investigated further. Despite the very limited study done in STP organisations, substantial work published related to ERP adoption need to be reviewed to strengthen and clarify the research focus. Additionally, the study foresees the ERP as a tool for enhancing business competitive advantage. Yet, technology cannot stand alone to promise success. It facilitates organisations to create capability and ability to succeed. Hence, it is suggested that the conceptual framework be revised. A revisit on the proposed theories is also recommended as the Balanced Scorecard is not a theory.

Are the rationale for, and objectives of, the Systematic Review clearly stated?

Partly

Is the statistical analysis and its interpretation appropriate?

Not applicable

Are sufficient details of the methods and analysis provided to allow replication by others?

Partly

Are the conclusions drawn adequately supported by the results presented in the review?

Partly

Reviewer Expertise:

Management Accounting and Control System

I confirm that I have read this submission and believe that I have an appropriate level of expertise to confirm that it is of an acceptable scientific standard, however I have significant reservations, as outlined above.
